# The effect of flipped classroom in multiple clinical skills training for clinical interns on Objective Structured Clinical Examinations (OSCE)

**DOI:** 10.1080/10872981.2021.2013405

**Published:** 2021-12-13

**Authors:** Weihao Zhang, Jiaoyang Gu, Fan Li, Feifei Feng, Huiqiang Chen, Xiaowei Xing, Lan Liu

**Affiliations:** a Department of Gastroenterology, The Second Hospital, Cheeloo College of Medicine, Shandong University, Jinan, Shandong, China; b Department of Neurology, The Second Hospital, Cheeloo College of Medicine, Shandong University, Jinan, Shandong, China; c Department of Respiration, The Second Hospital, Cheeloo College of Medicine, Shandong University, Jinan, Shandong, China; d Department of Cardiology, The Second Hospital, Cheeloo College of Medicine, Shandong University, Jinan, Shandong, China

**Keywords:** Flipped classroom, traditional classroom, Objective Structured Clinical Examination, clinical interns

## Abstract

**Aims:**

The flipped classroom (FC) is a hybrid approach, combining online learning and face-to-face classroom activities. To comprehensively evaluate the role of the Flipped Classroom (FC) model in clinical skills teaching of medical interns and investigate the acceptance and recognition of FC and Objective Structured Clinical Examinations (OSCE).

**Methods:**

In the teaching of clinical skills, the students were further grouped into two groups- A and B (A = 37, B = 42), using a computer-based random digital method. group A adopted the traditional classroom (TC) model, and group B adopted the FC model. OSCE was used to assess the clinical skills of the two groups of interns. Two independent sample t-test was used to analyze the difference of participant’s demographic data and OSCE scores between the two different teaching model. We sent FC questionnaires to group A and OSCE questionnaires to groups A and B.

**Results:**

The score of OSCE in group B was higher than that in group A (P = 0.024), especially in the heart physical examination (P < 0.050), lung physical examination (P < 0.050), and abdominal physical examination (P < 0.050). The result of the FC questionnaires showed that regarding online courses, most students in group B thought watching videos was a good way to prepare for class (97.6%), For offline courses, most medical interns said that it enhanced their learning ability (88.1%), and they could accept this form of teaching (85.7%). As for the form of OSCE questionnaires, most people in group A and B said that they knew more about this form of assessment (81.0%), that it truly reflected the clinical ability (82.3%).

**Conclusions:**

FC model has shown good results in clinical skills training, while FC and OSCE can be further promoted in future teaching and assessment.

## Introduction

The flipped classroom (FC), also called the inverted classroom [1], has become a leading teaching strategy and has a transitional effect on learners to change their learning styles [2]. In this new educational teaching method, learners learn the basic content independently before class, such as previewing the chapters to be taught in class in advance and watching a learning video on the online platform or multimedia presentation before class time [3]. The advantage of this online option is that it makes up for the lack of internal experts, and students can access courses online anytime and anywhere without geographical and time constraints [4]. A growing body of literature shows the FC to be an increasingly common educational strategy in medical education, with promising results in enhancing students’ performance in written exams [5,6]. FC can increase student participation in the courses, improve they awareness of course content, which provides impetus for students to revisit the material and thus helps to promote self- regulated behavior [7]. What’s more, it can improve performance on higher cognitive tasks, promote order problem solving and enhance clinical reasoning skills which is assessed by an OSCE [8]. In the background of clinical skills education, the use of the FC model has become more common in medical education. Studies have shown that it helps to improve students’ skills [9], increase students’ participation, and reduce the time required for teachers to teach [10]. Talking about the feeling of medical students, most people think that the FC is more flexible, what’s more, compared with TC, it has shorter teaching time and more opportunities to interact with the lecturer. The disadvantage is that it needs more time, but this is conducive to the strengthening of knowledge [11]. For clinical skills teaching, non-inclusion as part of the core curriculum for training, assessment without scores, or some students’ insufficient participation in the course and failing to keep up with the progress of the course will have an impact on the final training effect [12]. Objective Structured Clinical Examinations (OSCEs) have been established as a reliable assessment method of clinical aptitude and practical skills acquisition. As such, they now form a substantial part of the graduation process in numerous healthcare professions worldwide [13]. However, the impact on OSCE performance has been varied, with studies reporting either improvements or neutral effects [6,14], and the survey results on students’ test scores and satisfaction levels show mixed results in different studies [15]. Moreover, the effects of the FC in terms of changing the students’ skills are uncertain [16]. The uncertainty of the effectiveness of the FC and the heterogeneity in the published literature provide the impetus for the current research. In China, the pedagogic methods are mostly teacher-controlled didactic lecturing, the training modes of medical education are inflexible. Student-entered and competence-based medical education are not widely implemented. This study aims to evaluate the impact of FC on interns’ performance in various clinical skills assessments, and compare them through OSCE models. Investigating students’ views on FC and OSCE, which will provide a basis for the reform and implementation of FC education for clinical undergraduates in China.

## Materials and methods

In recent years, more attention has been paid to the teaching of clinical operation skills. Under the new medical education training mode, the teaching and training tasks and standards of clinical operation skills have been raised to a new height. Although the national opinions on education reform have been issued, there are still many problems in the front line of teaching in medical colleges and universities, such as using traditional teaching method, it is difficult to change the current situation of students’ strong theoretical learning ability and poor practical ability, and clinical work is urgent. There are contradictions in medical teaching, and trainee students are absent form class, these problems need to be solved urgently.

The exclusion criteria for our experimental interns is that they have previous clinical skills training experience, and no students were excluded. Part of the online video for this study comes from the People’s Medical Publishing House, a medical publishing institution and relevant teaching videos are not free and open, lasting 55 minutes (including heart, lung, abdominal, and neurological physical examinations). The online video of CPR were recorded by authoritative medical examination experts and lasted 7 minutes. The FC and OSCE questionnaires were produced by members of the research team. This is a prospective randomized controlled study on medical interns in the hospital. Before the formal trial, 79 interns were randomly divided into two groups- A and B (A = 37, B = 42), in computer-based random digital method. Group B participated in FC, and group A participated in TC. The online video was placed on a USB flash disk and distributed to the students in group B, not to group A, and the completion of the videos were obtained by the FC questionnaires. The offline courses of the two groups were taught by identical medical school professors. Before the beginning of the course, group B was randomly divided into 5 groups in the same way, and one of groups presented a report in the form of PPT. The reporting sequence and content were randomly assigned. The presentation time was controlled at 20 minutes, the group discussion time was 15 minutes, the review time was 10 minutes, and the total time was 45 minutes. Offline FC were regularly held every Monday, with a total of 5 times. Offline course content of group A was completed by weekly PPT teaching and course content summary, and the teaching time, frequency, total time and content of teaching were the same as that of group B. Offline clinical skills teaching was conducted in the afternoon after the the weekly offline courses, including 20 minutes of teacher skills teaching and 25 minutes of students practice, a total of 5 training sessions per group. The course composition is shown in (Table 1). The skills assessment was based on OSCE and was conducted on the third day after the end of all skills teaching. the location and materials were prepared by the hospital clinical skills examination center, and each station was equipped with a simulated person equipped with physical examination equipment. The offline teaching, clinical skills training and OSCE of the two groups were conducted separately.

**Table 1.** Course composition

	Group A	Group B
The number of online sessions	0	5
The number of offline sessions	5	SameasgroupA
Offline clinical skills teaching contents of sessions	Including heart, lung, abdominal, neurological physical examinations and CPR skills training.	Same as group A
Offline clinical skills teaching duration of sessions	Each teaching lasts 45 minutes per week, 5 times in total	Same as group A

We designed FC and OSCE questionnaires and used alpha to calculate the internal consistency and reliability of groups A and B. The alpha values of the OSCE questionnaire in group A and the OSCE and FC questionnaires in group B were 0.907, 0.925, and 0.888, respectively. The reliability of both questionnaires met the requirements. IBM SPSS Statistics 26 was used to analyze the two groups of questionnaires. We measured 4–9 items of the OSCE questionnaires and 4–14 and 18–21 items of the FC questionnaires according to the Likert scale of 1–5 (strong disagreement-strong agreement). We classified scores, in which ≥ 4 points are defined as consent. This study passed the Ethics Committee of the second Hospital of Shandong University (LCLL-2021-007).

### Procedures

All members of group B participating in FC had to complete the online videos 3 days before the start of the offline course. Before the course began, we randomly divided group B members into five groups, using a computer-based random digital method, and introduced the FC model to the subjects in group B in detail, so that they had a full understanding of the courses they were participation in. Group A also used the same grouping method. Each group were randomly assigned the content for physical examination and reported the selected content, using PPT format [17], and then freely discussed the difficulties and question encountered in online course learning. the teacher would make unified answers. After collating this group reports and comments, the teacher summarized the important and difficult points of this course. This offline course once a week. The teaching of group A comprised two parts: the weekly offline PPT teaching and the teacher’s review and summary of the content after class, and They didn’t have online videos.

### Offline clinical skills teaching

After finishing all the courses, Groups A and B were trained separately on what they had learned using the equipment of the Clinical Skills Center of this hospital. The training content, duration, and instructors of the two groups were the same.

### Skills assessment

**Figure 1.**
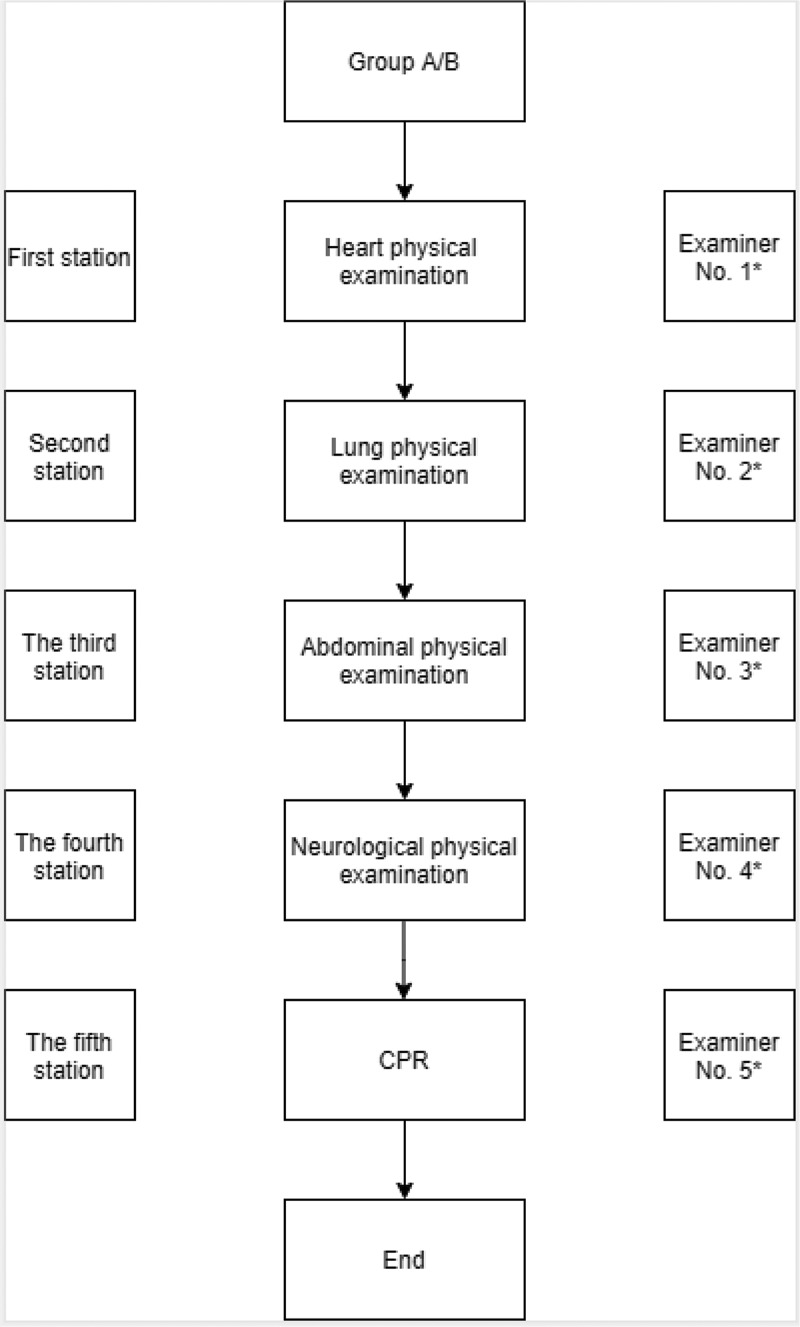
OSCE design

After clinical skills teaching, both groups A and B conducted OSCE at the Clinical Skills Center of this hospital. The assessment items comprised five stations: heart physical examination, lung physical examination, abdominal physical examination, neurological physical examination, and CPR. The assessment location of OSCE is the clinical skills training center of the hospital. There were 5 stations in OSCE, with 5 examiners, 1 from each station, all of whom have experienced professional training and participated in the national OSCE, and the examination form was revised by them. The order of stations is heart, lung, abdominal and neurological physical examination and CPR (Figure 1). There were no standard patients recruited, all were standard simulators provided by the hospital clinical skills center, and each station has an independent assessment space. All five stations refer to the national standardized training skills examination for interns. Before the examination, the relevant preparations passed the examination of the standardized training center for interns in this hospital.

*An OSCE professional examiner was set up at each station

### Questionnaires

After the OSCE, the instructor distributed anonymous OSCE questionnaires to groups A and B, and the FC questionnaires in group B were issued. Before the study, we obtained the informed consent of the subjects. The FC questionnaires included three basic contents of the subjects’ gender, age, current internship department, 15 scale questions, two multiple-choice questions, one other type of question, and one open question. It included five dimensions acceptance, recognition, participation, online course duration preference, and opinions of the FC. The content of OSCE questionnaires included three items of basic personal information, seven items of scale questions, and one item of an open-ended question, including three dimensions: acceptance, recognition, and suggestions of OSCE.

### Date collection

**Figure 2.**
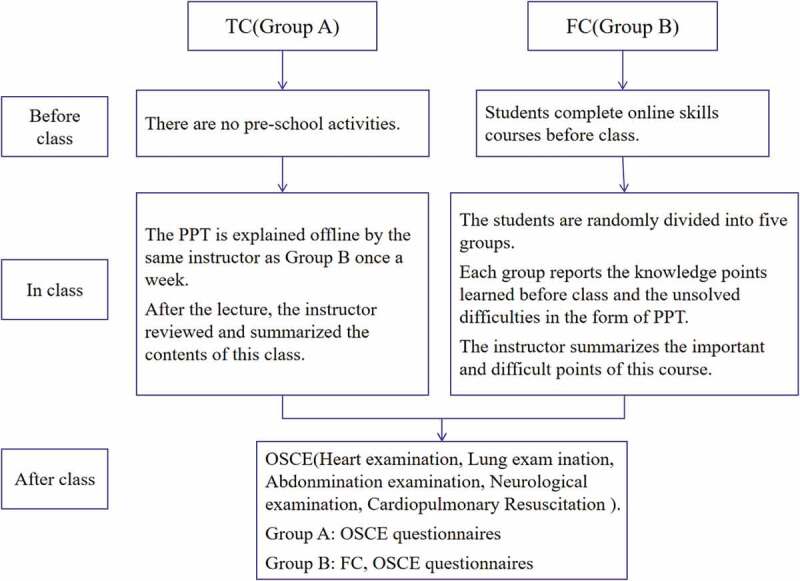
Experimental design

A total of 37 interns in group A participated in the TC and OSCE, and a total of 37 valid OSCE grade forms and questionnaires were collected. A total of 42 interns in group B participated in the FC and OSCE simultaneously, and 42 valid OSEC grade forms and OSCE and FC questionnaires were obtained (Figure 2).

### Statistical analysis

We used the Mac 2019 version of Microsoft Excel to collect all the OSCE assessment score data and the FC and OSCE questionnaire data. IBM SPSS Statistics 26 was used to test the normality and homogeneity of variance between groups A and B. Two independent sample t-test was used to analyze the difference of OSCE scores between the two different teaching model. P < 0.050 determined that it was statistically significant.

## Results

### Participants’ demographic data

The demographic data of group A and B are shown in (Table 2). This study included 79 clinical interns within two groups: A (n = 37) and B (n = 42). The average age of students in group A was 27.62 ± 2.95 years old, and that of students in group B was 27.57 ± 3.42 years old. 12 people (32.43%) in group A and 14 people (33.33%) in group B are married. All interns denied any work and flipped classroom course experience. There was no significant difference in sex (p = 0.945), marital status (P = 0.867) and average age (p = 0.379) between the two groups.

**Table 2.** Demographic data analysis

Characteristics	Group			
	A (n = 37)		B (n = 42)	
	Mean ± SD	N(%)	Mean ± SD	N(%)
Age (year)	27.62 ± 2.95		27.57 ± 3.42	
Gender(male/female)		9/28(24.32%/75.68%)		11/31(26.19%/73.81%)
Marital status		12(32.43%)	0	14(33.33%)
Experience of working		0		0
Flipped classroom course experience		0		0

Age is expressed as the means ± SD. Group B, flipped classroom; Group A, traditional classroom. *Independent sample t-test. **χ2-test.

### Results of OSCE

The average scores of all skills in groups A and B were in accordance with normal distribution and homogeneity of variance, as shown in (Table 3). There were significant differences in heart physical examination (96.670 ± 1.300 vs. 95.000 ± 2.380, P < 0.050), lung physical examination (96.830 ± 2.219 vs. 93.510 ± 1.938, P < 0.050), abdominal physical examination (96.550 ± 1.292 vs. 94.620 ± 1.831, P < 0.050), and average score of five groups of skills (95.748 ± 1.417 vs. 95.043 ± 1.283, P = 0.024). The results showed otherness at 0.05 significant level, and the average score of group B was higher than that of A. There was no difference in average skills scores between groups A and B in neurological physical examination (91.620 ± 4.818 vs. 93.050 ± 3.993, P = 0.157) and CPR (97.840 ± 1.424 vs. 97.880 ± 2.233, P = 0.920).

**Table 3.** Results of students’ skill examination

Group	T	p value
Group A_0_	2.304	0.024*
Group B_0_		
Group A_1_	3.923	<0.001*
Group B_1_		
Group A_2_	7.037	<0.001*
Group B_2_		
Group A_3_	5.451	<0.001*
Group B_3_		
Group A_4_	1.430	0.157
Group B_4_		
Group A_5_	0.101	0.920
Group B_5_		

* It indicates that there is a difference below the significance level of 0.05. A_0_ and B_0_ are the average skill scores of two groups, A_1_ and B_1_ are the average skill scores of heart physical examination, A_2_ and B_2_ are the average skill scores of lung physical examination, A_3_ and B_3_ are the average skill scores of abdominal physical examination, A_4_ and B_4_ are the average skill scores of neurological physical examination, A_5_ and B_5_ are the average skill scores of CPR.

### Interns’ perspectives survey about FC and OSCE

(Table 4) shows the results of the FC questionnaire. Regarding online courses, more interns thought that pre-class courses could be prepared for class (97.6%), and pre-class video learning resources were more suitable for them (90.5%), increasing their interest in learning (90.5%). Regarding offline courses, most interns said they liked the form of teacher-student interaction (90.5%) and thought that the discussion activities in class enhanced their learning ability (88.1%) and helped them gain a more comprehensive understanding of what they learned (92.8%). Regarding the FC model, most interns could accept this form of teaching (85.7%). They thought that FC could improve the performance of clinical skills (85.7%), which was more efficient than TC (88.1%). They were in favor of continuing the pilot project of the FC model in this hospital (83.4%).

**Table 4.** The method of questionnaire was used to investigate the feelings of FC teaching mode in group.(GroupA, B)

Content	5	4	3	2	1
Q1:I think studying the relevant courses before class can prepare for class.	31/42 (73.8%)	10/42 (23.8%)	1/42 (2.4%)	0/42	0/42
Q2:The study of pre-class video courses has increased my interest in learning.	22/42 (52.4%)	16/42 (38.1%)	4/42 (9.5%)	0/42	0/42
Q3:The video learning resources provided before class are very suitable for me.	24/42 (57.1%)	14/42 (33.3%)	4/42 (9.5%)	0/42	0/42
Q4:Compared to the online courses offered by the school, I prefer to find some learning materials by myself.	14/42 (33.3%)	12/42 (28.6%)	14/42 (33.3%)	1/42 (2.4%)	1/42 (2.4%)
Q5:I finished the pre-class video as required, but my extracurricular time is very short.	9/42 (21.4%)	14/42 (33.3%)	4/42 (9.5%)	10/42 (23.8%)	5/42 (11.9%)
Q6:I am glad to be watch the study video instead of the traditional Live lecture before class.	14/42 (33.3%)	13/42 (31.0%)	14/42 (33.3%)	1/42 (2.4%)	0/42
Q7:I really like this way of teaching in which teachers and students interact with each other in class.	23/42 (54.8%)	15/42 (35.7%)	4/42 (9.5%)	0/42	0/42
Q8:The discussion activities in class enhanced my learning ability.	19/42 (45.2%)	18/42 (42.9%)	5/42 (11.9%)	0/42	0/42
Q9:Through problem analysis and discussion in class, I have a more comprehensive and in-depth grasp of what I have learned.	24/42 (57.1%)	15/42 (35.7%)	3/42 (7.1%)	0/42	0/42
Q10:Compared with the TC, I think the FC is more conducive to the cultivation and improvement of medical students’ self-study ability.	21/42 (50%)	18/42 (42.9%)	3/42 (7.1%)	0/42	0/42
Q11:Compared with the TC teaching, I think the learning efficiency of the flipped classroom is higher.	20/42 (47.6%)	17/42 (40.5%)	5/42 (11.9%)	0/42	0/42
Q12:Compared with the TC, I think the FC can improve my clinical skills more obviously.	14/42 (33.3%)	22/42 (52.4%)	6/42 (14.3%)	0/42	0/42
Q13:I can accept the teaching form of FC.	17/42 (40.5%)	19/42 (45.2%)	6/42 (14.3%)	0/42	0/42
Q14:I am very satisfied with the teaching mode of FC this month.	15/42 (35.7%)	22/42 (52.4%)	5/42 (11.9%)	0/42	0/42
Q15:I agree that our hospital should continue to carry out the pilot project of FC teaching.	17/42 (40.5%)	18/42 (42.9%)	7/42 (16.7%)	0/42	0/42

point Likert responses(5 = strongly agree, 1 = strongly disagree)collected via Score table.

(Table 5) shows the results of the OSCE questionnaires. Most interns said they knew more about the OSCE model (81%) and thought that it truly reflected the doctor’s clinical ability (82.3%). Moreover, it helped to improve the doctor’s comprehensive ability (95%). Most of them thought that this model was more novel, fair, and objective than the traditional assessment model (86%) and liked this assessment model significantly (87.4%). They thought that it was necessary to promote it in the entrance examination of doctors (81%). In terms of the difficulty of skill examination, the selection rate of neurological physical examination was the highest (40.5%), followed by lung physical examination (32.9%), heart physical examination (13.9%), no difficulty (13.9%), and abdominal physical examination (5.1%) (Figure 6) The selection rate of CPR was the lowest (2.5%).

**Table 5.** The method of questionnaire was used to investigate the feelings of OSCE of the two groups of trainee medical students.(Group A, B)

Content	5	4	3	2	1
Q1:I know a lot about OSCE.	26/79 (32.9%)	38/79 (48.1%)	8/42 (10.1%)	6/42 (7.6%)	1/42 (1.3%)
Q2:I think the OSCE is beneficial to the improvement of doctors’ comprehensive ability.	39/79 (49.4%)	36/79 (45.6%)	3/42 (3.8%)	0/42	1/42 (1.3%)
Q3:I think the OSCE teaching evaluation model can truly reflect the clinical ability of doctors.	30/79 (38.0%)	35/79 (44.3%)	11/42 (13.9%)	2/42 (2.5%)	1/42 (1.3%)
Q4:Compared with the traditional examination, OSCE is more novel, fair and objective.	31/79 (39.2%)	37/79 (46.8%)	9/42 (11.4%)	1/42 (1.3%)	1/42 (1.3%)
Q5:I like this mode of teaching evaluation very much.	30/79 (38.0%)	39/79 (49.4%)	9/42 (11.4%)	1/42 (1.3%)	0/42
Q6:I think it is necessary to extend the OSCE teaching evaluation model to the entrance examination of doctors.	32/79 (40.5%)	32/79 (40.5%)	12/42 (15.2%)	2/42 (2.5%)	1/42 (1.3%)

**Figure 3.**
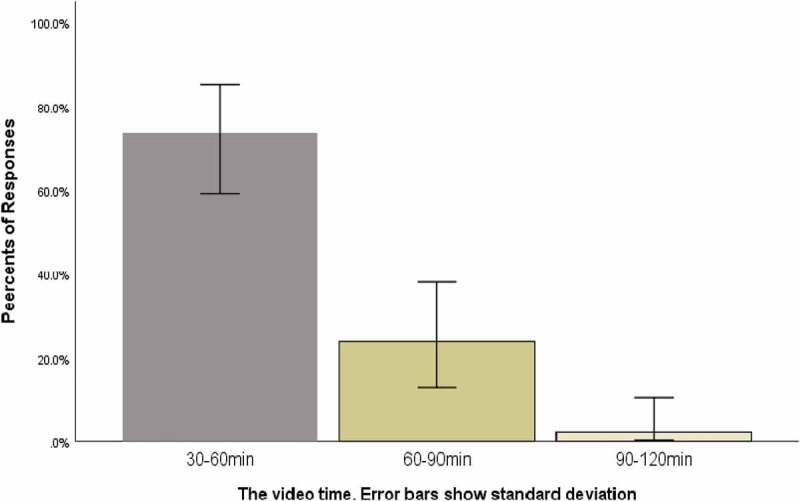
How long do you think it is appropriate to control the duration of self-study video before class?(Group B)

**Figure 4.**
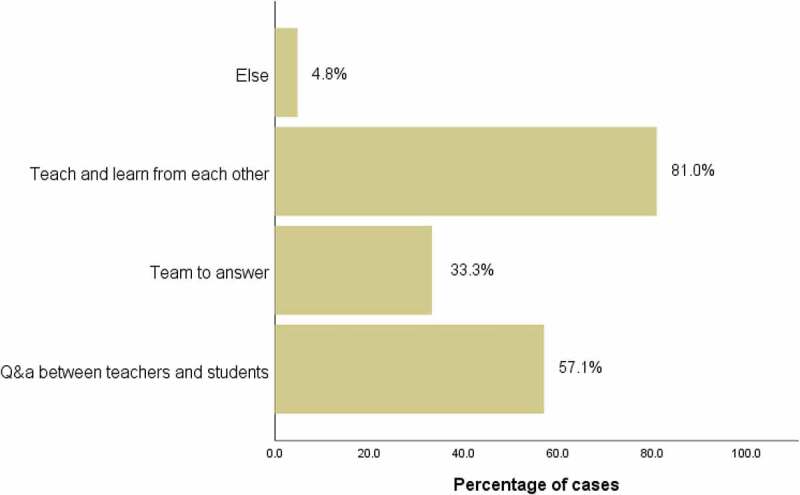
What do you think of a valid interactive communication method in class?(Group B)

**Figure 5.**
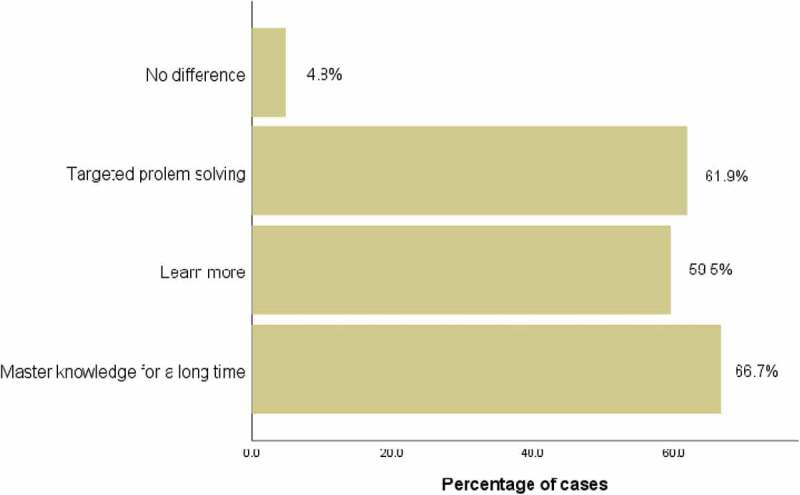
Compared with traditional classroom teaching, what do you think of the learning effect of FC?(Group B)

Most of them thought it was appropriate to limit the video duration to 30–60 minutes (73.8%) (Figure 3). Most students believed that the effective way of interaction in class was teaching and learning from each other (81.0%) (Figure 4), and FC could solve problems more specifically (61.9%), which was conducive to the long-term mastery of knowledge (66.7%) (Figure 5).

**Figure 6.**
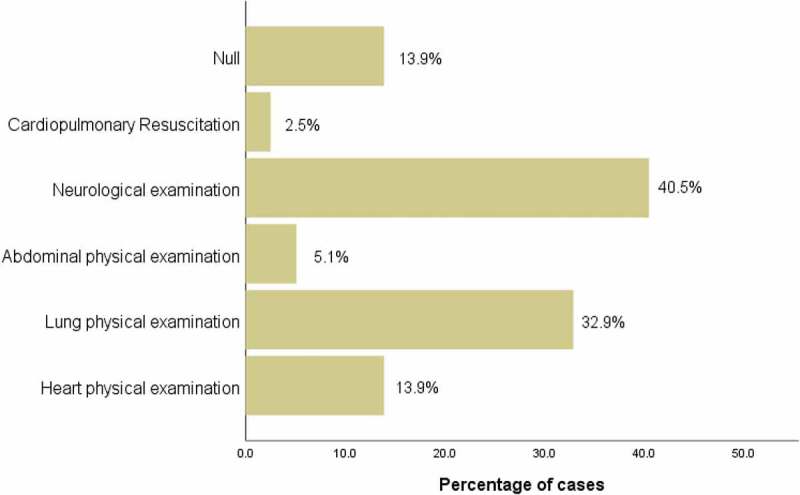
Which operation or inspection do you think is difficult in this assessment?(Group A, B)

## Discussion

The purpose of this study is to evaluate the role of FC in clinical skills training, so as to provide a basis for the reform of medical education in China. The result of our study showed that interns performance significant improved with the FC approach in heart, lung and abdominal physical examination, which were consistent with those in physical examination skills for radiology [18], osteopathic [19], laparoscopic suturing skills [20] and surgical competence [21]. However, there was no significant difference in OSCE scores between the two groups in the neurological examination, with the lowest average score of all grades. A study have shown that medical students have neurophobia, which has been demonstrated throughout the world, and neurology has the complex neurologic examination, difficult neuroanatomy and poorly taught neurology curricula [22]. Based on this we speculate that the interns were nervous about the high difficulty of neurological physical examination and the lack of teaching, which affected their assessment results. Adding 3D neurological and video demonstration could optimize the teaching effect of neurological physical examination and alleviate this anxiety [23]. Some results of our study are inconsistent with previous literature, which is not surprising given the wide range of results from previous studies. One study compared the written test results of the FC and TC models for learning Advanced Cardiac Life Support (ACLS). The results showed that score of the FC group was higher than that of the TC group [24]. In this study, there was no statistical difference in CPR scores between groups A and B. Previous studies have shown that the scores of both groups were close to 100 and that there was interference between each other in the course of the study [14]. In our study, the scores of CPR in both groups were considerably high, which may resulted in no significant difference between the scores of the two groups. In the future, we will consider adding the content of CPR assessment on the original basis to increase the degree of distinction.

The reason why FC improves the performance of clinical skills may it offers the opportunity for students to engage in higher order cognitive activities such as analysis, evaluation and synthesis of knowledge, and result in improvement in higher order problem solving and clinical reasoning. In the FC questionnaires, most interns thought that online courses were a good way to prepare before class, and previous studies had similar conclusion [12]. A study showed that the FC model increased their interest in learning because it allowed students to think independently and find answers through grouping, a process that stimulates learning motivation [25]. Analyzing the reasons why pre-recorded videos increase the enthusiasm for learning, researches has shown that they enable students to arrange their study plants at their own place, regardless of time and place. For the knowledge points that were not well mastered, students could broadcast them many times according to the actual situation, which would not only improve their satisfaction with teaching but also allow them to have flexible rest according to their needs [26–28]. In our study, some students reflected that the extracurricular time was considerably short. We speculate that the reason for this situation is the excessive work intensive and pressure during the internship. Consequently, there was insufficient time to understand what their learned [12], The emergence of this problem requires the relevant education departments to reform the internship program to better improve the quality of teaching. It can be seen from the results of the FC questionnaire that most students can accept a short online video, and we concluded that when teaching content is presented in a shorter video length, content organization and viewer satisfaction improve. Our conclusions agree with previous study in which comparing video length and the inclusion of supporting content [8], mentioned that materials that convey key concepts quickly in a short period of time are preferable to materials that must be sorted and digested slowly [8]. For this we infer shorter videos are more suitable for students who are internships in hospitals because of their tight time and more conducive for them to integrate materials into the background of the entire course. As for offline courses, most interns said that they liked the form of teacher-student interaction, and the previous article also reached a similar conclusion [25]. Although most interns thought that they could accept the FC, the students who had just come into contact with it needed a certain period of adaption, during which they could be familiar with and accept it [29]. From the FC questionnaire, we can conclude that quite a number of interns have a positive and receptive attitude towards FC, which also provides a good basis for the wider implementation of this teaching form in China in the next step.

Since its creation in 1975, OSCE has been recognized as an effective assessment tool and has been implemented by many educational institutions to assess the clinical performance of students [30]. Students believed that OSCE was beneficial to the improvement of doctors’ comprehensive ability. A study have also emphasized that students believed that participating in OSCE was inspiring and a good learning opportunity [31]. There were still some students said they were not well prepared for the exam, which would increase their anxiety during the examination and reduce the clarity of their own evaluation. So the form of OSCE game could be adopted to reduce stress and improve their time management ability [13]. It can be concluded from the questionnaire that most of the interns can accept the skill assessment model of OSCE, and we can also predict that this model will have a good acceptance in the future.

Like all the studies on educational practice, our research has multiply shortcomings. First of all, we put the online videos on a USB flash disk and distributed it to the interns to watch and learn on their own, which made us unable to know and analyze the student’s habit of watching the videos, such as double speed or slow down, nor can we accurately grasped the number of times the students watched. We could only get the completion of the videos from the questionnaires. In the future, we will upload the learning video to a large video transmission platform like Tencent Videos, and consider developing further means of measuring student participation and adherence to the flipped classroom protocol. This could be accomplished through administration of a student survey, where students can anonymously report whether they insist on watching instructional videos [19]. Secondly, online and offline courses were not the same lecturer, we can not control the quality of lecturer teaching, some teachers with strong ability will be more attractive to students, which may lead to different teaching quality of each class. In the future, we will quantity the lecture’s teaching quality in advance to reduce the interference of this factor to the research results.

In conclusion, compared with the TC, the FC model may be a better choice in teaching clinical skills of medical interns. The FC model can stimulate interns’ learning motivation, enhance their learning ability, make their knowledge more comprehensive and in-depth, and improve their performance in the OSCE. The FC model needs to be more widely implemented in more clinical skills training and further optimized in the undergraduate graduation evaluation system.
